# Unveiling GATA3 Signaling Pathways in Health and Disease: Mechanisms, Implications, and Therapeutic Potential

**DOI:** 10.3390/cells13242127

**Published:** 2024-12-22

**Authors:** Rim Bacha, Nouran Alwisi, Rana Ismail, Shona Pedersen, Layla Al-Mansoori

**Affiliations:** 1 College of Medicine, QU Health, Qatar University, Doha P.O. Box 2713, Qatar; rb1201900@student.qu.edu.qa (R.B.); na2104730@student.qu.edu.qa (N.A.); ri2108210@student.qu.edu.qa (R.I.); 2 Biomedical Research Center, Qatar University, Doha P.O. Box 2713, Qatar; 3 College of Health Sciences, Qatar University, Doha P.O. Box 2713, Qatar

**Keywords:** GATA3 transcription factor, cell differentiation, cancer biomarker, cell proliferation, apoptosis, immune regulation, therapeutic implications

## Abstract

GATA binding protein 3 (GATA3), a member of the GATA family transcription factors, is a key player in various physiological and pathological conditions. It is known for its ability to bind to the DNA sequence “GATA”, which enables its key role in critical processes in multiple tissues and organs including the immune system, endocrine system, and nervous system. GATA3 also modulates cell differentiation, proliferation, and apoptosis via controlling gene expression. In physiological instances, GATA3 is crucial for maintaining immunological homeostasis by mediating the development of naïve T cells into T helper 2 (Th2). In addition, GATA3 has been demonstrated to play a variety of cellular roles in the growth and maintenance of mammary gland, neuronal, and renal tissues. Conversely, the presence of impaired GATA3 is associated with a variety of diseases, including neurodegenerative diseases, autoimmune diseases, and cancers. Additionally, the altered expression of GATA3 contributes to the worsening of disease progression in hematological malignancies, such as T-cell lymphomas. Therefore, this review explores the multifaceted roles and signaling pathways of GATA3 in health and disease, with a particular emphasis on its potential as a therapeutic and prognostic target for the effective management of diseases.

## 1. Introduction

GATA3, a member of the GATA family of transcription factors, plays a vital role in regulating gene expression and key cellular processes, including differentiation, proliferation, and apoptosis. It is essential for the development and maintenance of various tissues and systems, including the immune, endocrine, and nervous systems [1,2]. Dysregulation of GATA3 is implicated in several diseases, such as breast cancer (BC), hematological malignancies like T-cell lymphomas, autoimmune diseases, and neurodegenerative disorders [1,2]. In cancer, GATA3 influences tumor development and progression by affecting cancer cell behavior within the tumor microenvironment (TME) [3,4]. For instance, reduced GATA3 expression in breast cancer is linked to a poorer prognosis. In autoimmune diseases such as multiple sclerosis, abnormal GATA3 expression disrupts immune system balance, contributing to the autoimmune attack on the nervous system [5]. In neurodegenerative disorders like Alzheimer’s disease, GATA3’s role in neuronal function and maintenance suggests that its dysregulation may accelerate disease progression [6,7].

GATA3’s involvement in these conditions highlights its potential as a biomarker for diagnosis, prognosis, and personalized treatment strategies. Its altered expression patterns provide insights into disease pathology and patient outcomes, making it a valuable tool in clinical practice [8,9]. Furthermore, GATA3 analysis helps uncover the shared genetic architecture between conditions like cancer and autoimmune diseases, guiding therapeutic interventions and disease management [10,11]. Overall, GATA3 stands out as a promising biomarker with significant implications for future research and medical applications.

## 2. GATA3 in Normal Physiology

### 2.1. Immune System Development

GATA3, a dual zinc finger transcription factor, is a critical modulator of the growth and differentiation of immune cells, thereby influencing their function in the body’s defense system [2,12]. Its significance is not restricted to specific cell types, as it impacts the biological processes of various cell types of both innate and adaptive immunities. GATA3 is abundantly found in the hematopoietic compartment, where immune cells evolve and differentiate into multiple types with distinct roles [2,12].

#### 2.1.1. Th2 Differentiation

GATA3 is essential for naive CD4+ T cells’ differentiation into Th2 cells. It integrates humoral mediators like interleukin-2 (IL-2) and interleukin-4 (IL-4), via Wnt and Notch signaling pathways, stabilizing Th2 function independently of cytokine stimulation [2,12].

#### 2.1.2. Other Immune Cells

While GATA3 is well known as a key determinant of Th2 cells, via integrating different upstream signals to regulate target genes, which differentiate into the helper CD4+ T-cell subset, emerging evidence suggests that it also plays a role in maintaining the differentiation and function of other T-cell subsets, as well as B cells and natural killer (NK) cells [2,12]. Moreover, GATA3 expression is upregulated in CD4 T cells and innate lymphoid cells (ILCs) offering an additional role in immunoregulation via distinct genetic programs based on cellular and micro-environmental contexts [2,12]. Strikingly, GATA3 possesses its own activation machinery apart from cytokine-dependent stimulation, enabling its stabilization in Th2 cells to maintain their function [2,13].

### 2.2. Endocrine System

#### 2.2.1. Mammary Gland Development

GATA3 is a fundamental mediator for cell fate processes and the mammary gland is a ductal epithelial organ comprising two main mature epithelial cell types (luminal epithelial and myoepithelial); thus, the role of GATA3 in the growth and differentiation of mammary glands has been the subject of speculation in various studies [14,15]. GATA3 regulates the morphogenesis, branching, and elongation of the mammary gland [14]. Deleting GATA3 selectively in the mammary epithelium during puberty using MMTV-Cre92 failed in the development of terminal end buds (TEBs) and the formation of abnormal ductal structures [1,14,15]. This suggests that GATA3 plays a role in tumorigenesis and breast cancer initiation by influencing the differentiation of both epithelial and non-epithelial tissues [15].

#### 2.2.2. Adipogenesis Regulation

Remodeling the white adipose tissue (WAT) at early preadipocyte differentiation can hinder adipogenesis [16]. Remodeling factors, such as GATA2 and GATA3, inhibit adipogenesis through direct binding to the Peroxisome proliferator-activated receptor γ (PPARγ) promoter, suppressing its activity and antagonizing CCAAT/enhancer binding protein α and β (C/EBPs) [17]. The continuous expression of GATA3 impedes adipogenesis by maintaining cells in the preadipocyte stage, with GATA3 levels gradually decreasing as adipocytes differentiate [18]. Targeted therapies for obesity and insulin resistance (IR) have been speculated based on outcomes of silencing GATA3 via specific DNAzyme, which induces adipogenesis in 3T3-L1 mouse pre-adipocytes [19]. Therefore, GATA3 implementation enhances adipogenesis, modifies fat distribution, improves insulin sensitivity, and reduces obesity-related inflammation, supporting its therapeutic relevance in type 2 diabetes (T2D) pathology [19,20].

### 2.3. Central Nervous System (CNS)

#### Neural Development, Neuroprotection, and Regeneration

GATA3 is a key player in neural development and differentiation as its expression is linked to early motor neurons and interneuron precursors, underscoring its pivotal involvement in the early developmental stages of the CNS [21]. Mice embryos with GATA3 mutations were fatal, exhibiting symptoms like internal bleeding, growth delay, abnormalities in fetal liver hematopoiesis, brain deformities, and spinal cord damage [22]. Moreover, blocking GATA3 activity depletes the ability of neural stem cells (NSCs) in the telencephalon of zebrafish to proliferate and generate neurons [23]. After injury, GATA3 expression is reactivated in glial cells and newly formed neurons, demonstrating its involvement in reactive neurogenesis and the migration of neurons [23].

### 2.4. Renal System

#### 2.4.1. Developmental Role

At renal developmental stages, GATA3 is predominantly found in the epithelial nephric duct of the renal primordium, where it regulates the positioning of the ureteric bud within the renal system [24]. Grote et al. [25] used a HoxB7-Cre transgenic line to specifically eliminate GATA3 from the nephric duct resulting in premature differentiation and formation of misplaced metanephric kidney ducts. Renal dysplasia has been linked to GATA3 insufficiency as well, revealing the substantial role of GATA3 in renal cell development [26].

#### 2.4.2. Cell Identity and Dysregulation

**Figure 1:**
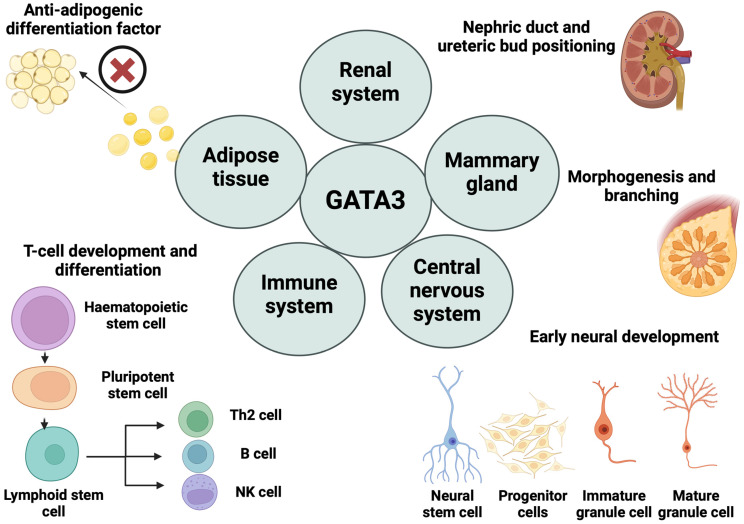
Diverse roles of GATA3 in cell differentiation and systemic development. This diagram illustrates several roles of GATA3 across different cell types and biological systems. GATA3 influences various processes, including adipogenesis, where it acts as an anti-adipogenic factor by inhibiting adipocyte differentiation. In the renal system, GATA3 is crucial for the positioning of the nephric duct and ureteric bud. It also plays a key role in the immune system, regulating hematopoietic, pluripotent, and lymphoid stem cells through Wnt and Notch signaling, leading to the differentiation of NK cells, T lymphocytes (Th2), and B lymphocytes. In the mammary gland, GATA3 is involved in morphogenesis and branching, essential for tissue development. Additionally, GATA3 contributes to early neural development in the central nervous system, influencing the differentiation of neural stem cells, precursor cells, and mature glial cells. (Drawn by biorender).

Additionally, renin cells’ lineage identity, having precursors in renal tissues, is strongly associated with GATA3 levels depending on previous research [27]. Mice with conditional knockout of GATA3 (GATA3-cKO) displayed remarkably diminished GATA3 in juxtaglomerular, mesangial, and smooth muscle cells, implying massive elimination of GATA3 in renin lineage cells. These GATA3-cKO mice displayed a notable rise in blood urea nitrogen levels, suggesting hypovolemia and/or impaired renal function [27]. Overall, GATA3 acts as a vital mediator for maintaining the biological processes and homeostatic functions for optimal regulation of the dynamism and productivity of living cells (Figure 1).

## 3. GATA3 in Pathological Conditions

### 3.1. Cancer

#### 3.1.1. Breast Cancer (BC)

**Figure 2:**
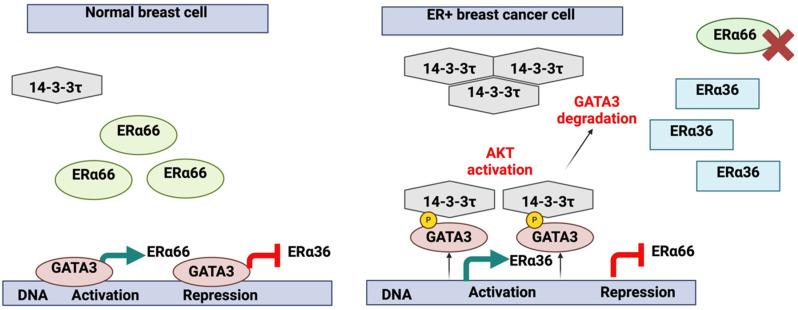
Molecular mechanisms controlling ERα36 transcription in normal versus luminal breast cancer cells. In normal cellular conditions, GATA3 binds to both ERα66 and ERα36 promoters, inducing ERα66 transcription while suppressing ERα36 transcription. However, during the evolution of breast cancer, certain ER+ breast cancer cells may acquire both high 14-3-3τ expression and aberrant AKT activation, either through intrinsic factors or interactions within the TME. These incidents result in GATA3 phosphorylation, facilitating its interaction with 14-3-3τ, hence losing its transcriptional control over ERα66 and ERα36. Therefore, ERα36 is transcribed and opposes the expression of ERα66, leading to the downregulation of ERα66 and the acquisition of more basal-like cell characteristics. ERα36, estrogen receptor alpha 36; ERα66, estrogen receptor alpha66; AKT, protein kinase B; TME, tumor microenvironment. (Adapted from Garan et al. [28] and re-drawn by biorender).

The eligibility of GATA3 as a key marker in BC diagnostics and prognostics is attributed to close association with ER status (Figure 2).

#### 3.1.2. Luminal Differentiation and Tumor Progression

**Figure 3:**
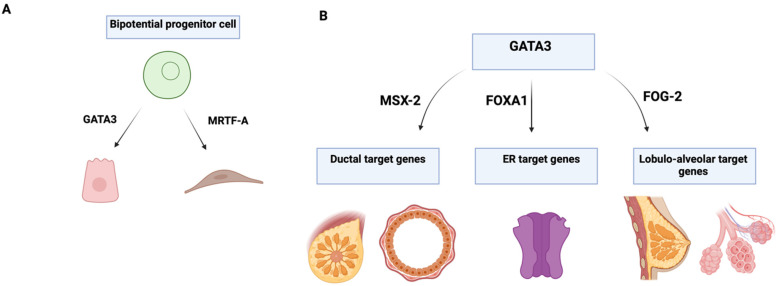
(**A**) Representation of GATA3 in maintaining the luminal cell fate in the mammary gland while MRTF-A drives myoepithelial cell differentiation. (**B**) A streamlined GATA3-mediated gene regulatory network illustrating the established connections between GATA3 and other transcriptional regulators in the mammary gland. FOXA1 is a downstream effector of GATA3 signaling, MSX-2 acts as an essential modulator with GATA3 for mammary duct development. FOG-2 is a cofactor of GATA3, upregulated in pregnancy and lactation, and its interaction with GATA3 is highly required for lobulo-alveolar development. MRTF-A, myocardin-related transcription factor A; FOXA1, forkhead box protein A; MSX-2, Msh Homebox 2; ER, estrogen receptor; FOG-2, friend of GATA3-2. (Modified from Kouros-Mehr et al. [15] and re-drawn by biorender).

GATA3 is essential for maintaining luminal cell differentiation in the mammary gland [15], opposing the basal differentiation regulated by SOX10 [29]. GATA3 plays a key role in the morphogenesis and regulation of mammary epithelial differentiation, serving as an important tissue-specific marker for identifying whether tumors originate from epithelial or mesenchymal cells (Figure 3).

In triple-negative breast cancer (TNBC), which is known for its aggressiveness and poor prognosis, the absence of GATA3 expression suggests that some TNBC subtypes may lose their specific markers as progenitor cells transition into tissues with distinct morphologies, thereby increasing tumor aggressiveness [29]. While GATA3 is generally associated with luminal differentiation and better prognosis in breast cancer, some studies have reported conflicting results regarding its role in different subtypes. For instance, GATA3 expression is typically low in TNBC, which is associated with poor prognosis [30]. However, some rare TNBC subtypes may still express GATA3, complicating the understanding of its role in these cancers [29].

#### 3.1.3. Tumor Microenvironment (TME) and Macrophage Infiltration (MI)

Previous studies have demonstrated that the tumor microenvironment (TME) has a significant impact on GATA3 expression [31]. Elevated MI within the TME has been shown to reduce GATA3 levels, correlating with advanced tumor grade and poorly differentiated forms of BC [31]. Tumor-associated macrophages (TAMs) of the M2 phenotype promote tumor progression, further implicating the role of GATA3 in tumor differentiation and aggressiveness [31]. This finding accentuates the importance of the TME in modulating GATA3 expression and its potential impact on tumor progression. GATA3 influences several molecular pathways in BC. It binds to ERα66 and ERα36 promoters, regulating their transcription. During BC progression, aberrant activation of protein kinase B (AKT) and high 14-3-3τ expression led to GATA3 phosphorylation, disrupting its transcriptional control and promoting a basal-like cell phenotype (Figure 2) [28]. The potential of targeting GATA3 for therapeutic purposes is promising, but there are significant challenges. For example, the mechanisms underlying GATA3 downregulation in aggressive breast cancers are not fully understood, which complicates the development of targeted therapies. Moreover, the potential side effects and off-target impacts of modulating GATA3 expression need to be carefully evaluated in preclinical and clinical studies [32].

#### 3.1.4. GATA3 Is a Potential Diagnostic and Prognostic BC Marker

GATA3 serves as a powerful diagnostic marker for BC, outperforming traditional markers like gross cystic disease fluid protein 15 (GCDFP-15) and mammaglobin due to its stronger positivity and consistency in both primary and metastatic tumors [33,34]. Its expression is positively linked to ER levels, indicating a better response to hormonal treatments in patients with high GATA3 expression [35]. Although GATA3 is a powerful diagnostic marker for breast cancer [36,37,38,39,40], its utility may be limited by the heterogeneity of breast cancer subtypes. The variability in GATA3 expression across different subtypes and stages of breast cancer suggests that it may not be universally applicable as a prognostic marker [38]. Additionally, the reliance on GATA3 expression alone may not provide a complete picture of the tumor’s behavior and prognosis.

### 3.2. Leukemia and Lymphomas

#### 3.2.1. Genetic Variants and Risk Associations

The pathogenesis of hematopoietic malignancies, including leukemias, is potentially linked to inherited germline or somatic genetic variations [41]. While somatic genomic aberrations such as mutations and rearrangements drive the progression of leukemia, via maintaining the pro-survival of early-stage leukemia precursor cells, the inherited risk variants and their causative mechanisms in leukemogenesis remain poorly understood.

Upon ATAC-seq performed in a recent study, enriched GATA3 binding sites were in close approximation with the translocation breakpoints seen in Philadelphia chromosome-like acute lymphoblastic leukemia (Ph-like ALL) [41]. GATA3 overexpression was correlated potentially to the chromosomal instability and vulnerability to translocations that take place in earlier stages of cancer onset. Thus, GATA3 upregulation in leukemic patients might facilitate the enhancer seizing those results in closer rearrangement to the oncogenes, triggering oncogenesis without forming gene fusions.

**Figure 4:**
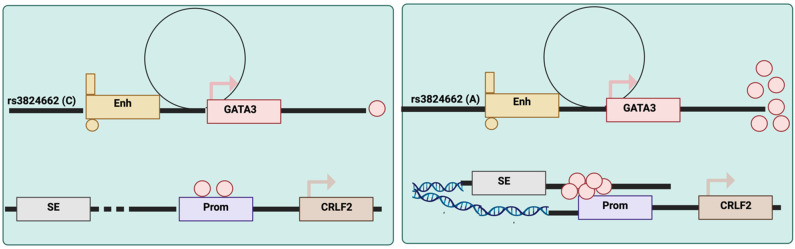
GATA3 potentiates CRLF2-JAK-STAT signaling in hematopoietic cells. A schematic model of the role of the rs3824662 variant in the pathogenesis of Ph-like ALL. The A allele stimulates *GATA3* expression, which binds to the *CRLF* promoter and loops the *CRLF2* promoter to the super enhancer at the *P2RY8* locus, eventually resulting in *CRLF2* overexpression. The chromatin region between the *CRLF2* promoter and *P2RY8* super enhancer also becomes more accessible, increasing its susceptibility to damage (e.g., rearrangements). Ph-like ALL, Philadelphia chromosome-like acute lymphoblastic leukemia; Enh, enhancer; SE, super-enhancer; Prom, promoter. (Adapted from Yang et al. [41] and re-drawn by biorender).

Recent research has identified noncoding genetic variations in GATA3 that increase the risk of acute lymphoblastic leukemia (ALL). For example, the rs3824662 variant acts as a cis-acting enhancer that accelerates GATA3 transcriptional activity, reshaping global chromatin accessibility and increasing susceptibility to chromosomal rearrangements [41], leading to increased incidence at regulatory sites and de novo occupancy in certain elements. Such a shift in the overall chromatin state plays a role in the development and progression of Ph-like ALL [41]. This discovery provides insights into the genetic regulation of GATA3 and its role in leukemogenesis (Figure 4).

Elevated GATA3 expressions have been detected in B-cell malignancies, including Hodgkin lymphoma, as well. The constitutive induction of NFkB and Notch-1 led to an aberrant increase in GATA3 levels within Reed–Sternberg cells accompanied by a massive amount of IL-13, ending up with typical signaling of Hodgkin lymphoma [42]. GATA3 is not present in normal B cells and mainly functions as a key regulator of lymphoid cell lineage commitment [43]. Collectively, it has been indicated that GATA3 is a major driver of epigenomic reprogramming that can directly influence the activity of oncogenes, suggesting a critical pathway through which inherited genetic variations might impact the risk of cancer [41].

#### 3.2.2. Oncogenic Role in T-Cell Lymphoproliferative Neoplasms

Interestingly, GATA3 activity is not limited to regulation and reprograming seen in genetically and clinically unique groups of T-cell lymphoproliferative neoplasms, as recent work has provided the first direct evidence that GATA3 is, indeed, a bona fide proto-oncogene that contributes to the aggressiveness for these neoplasms [44]. The oncogenic driving capacity of GATA3 originated from the induction of the transcriptional programs that promote cell growth and proliferation; hence, divergent enhancer landscapes were detected among GATA-3-associated T-cell neoplasms with different target genes [44]. Most of these genes were linked to T-cell lymphomagenesis and chemotherapy resistance [45], explaining the notable connection between GATA3 expression and adverse outcomes after receiving standard chemotherapy treatments [44]. This finding stresses the potential of targeting GATA3 in therapeutic strategies for T-cell lymphomas.

### 3.3. Liver Carcinoma

**Figure 5:**
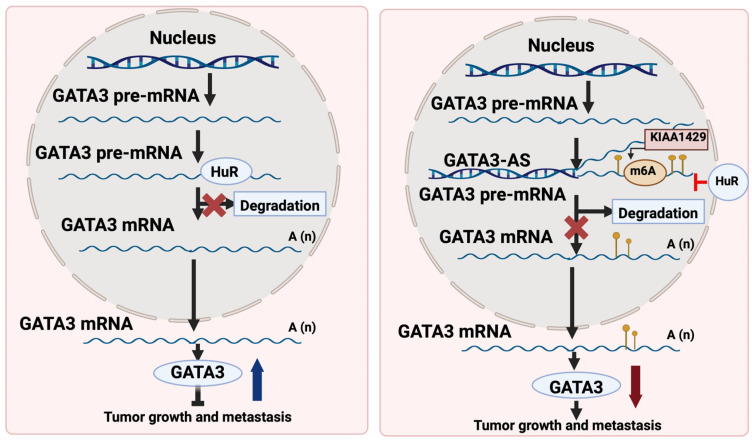
Schematic model of the epi-transcriptomic regulation underlying the KIAA1429-GATA3 pathway based on m6A modification. In normal liver cells (**left panel**), the interaction between HuR and GATA3 pre-mRNA remained stable, ensuring the persistence of GATA3 expression at relatively high levels. Conversely, in liver cancer cells (**right panel**), KIAA1429 methyltransferase, guided by GATA3-AS, selectively facilitated m6A methylation on the 3′ UTR of GATA3 pre-mRNA. This led to the dissociation of HuR from GATA3 pre-mRNA and subsequent degradation, resulting in downregulated expression of GATA3. HuR (Human antigen R) acts as an RNA-binding protein. GATA3-AS, GATA3 antisense strand; m6A, N6-methyladenosine. (Adapted from Lan et al. [46] and re-drawn by biorender).

GATA3 functions as the main activator for tumor suppressor genes via binding to their promoters. Nonetheless, the mechanisms behind GATA3 downregulation leading to switching off the tumor suppressor genes in most cancers remain elusive. One of the possible mechanisms of GATA3 depletion in hepatocellular carcinoma (HCC) is the post-transcriptional modification that disturbs its mRNA levels, thereby diminishing its protein levels [46]. In a previous study, GATA3 downregulation was attributed in HCC to the formation of GATA3-antisense (GATA3-AS) that antagonizes GATA3 and functions as a cis-regulatory element, thus directing m6A modification by KIAA1429 on the GATA3 pre-mRNA [46]. This post-transcriptional modification leads to the degradation of GATA3 mRNA, contributing to HCC progression [46]. On the contrary, GATA3-AS depletion was associated with suppressed malignant phenotypes in hepatoma cells that were rescued by GATA3 inhibition [46]. Understanding this novel mechanism of GATA3 downregulation in HCC opens new avenues for therapeutic interventions targeting GATA3-AS and m6A modification. GATA3 mediates the metastasis driven by KIAA1429 or GATA3-AS as shown in Figure 5.

Additionally, tumor suppressiveness of GATA3 was detected in HCC through its direct regulation of slug expression [47], thereby interfering with the EMT process, cell proliferation, invasion, and migration. Such a finding represents GATA3 as a promising candidate for designing therapeutic strategies against HCC.

#### 3.3.1. Prostate Cancer (PCa)

An antagonizing role of GATA3 in preventing the progression of prostate cancer has been determined in prior research [48]. The enforced GATA3 expression in prostate tumors interferes with Akt signaling and maintains a differentiated prostatic duct phenotype that is predictive of low tumor recurrence [48]. The latter work exhibited the potential of using GATA3 anti-oncogenic activity via regulating PI3K-Akt signaling as a prognostic biomarker for prostate cancer. Although GATA3 serves as a practical immunohistochemical marker for differentiating cancers with urothelial origins from prostate cancers, GATA3 positivity in metastatic prostate PCa may infrequently lead to misdiagnosis, necessitating a panel of immunohistochemical markers, for both prostatic and urothelial cancers and primary and metastatic tumors [49].

#### 3.3.2. Bladder Cancer

A loss of GATA3 function was consistently seen in high-grade invasive bladder cancer, suggesting the utility of GATA3 as a prognostic biomarker in addition to its utility in diagnostic procedures [50]. In general, a depleted level of GATA3 suggests a basal subtype of bladder cancer that is more sensitive to immune checkpoint blockade therapy (ICB) and neoadjuvant treatments. Conversely, upregulation of GATA3 refers to a luminal subtype of bladder cancer, exhibiting better response to targeted treatments, including a knockout of GATA3, β-catenin, and PPAR-γ pathways, as well as anti-angiogenic therapy. Therefore, the employment of GATA3 in the determination of TME phenotypes and bladder cancer subtypes has focused on GATA3 as a promising candidate for personalized medicine for better management of bladder cancer [51]. GATA3 is a plausible diagnostic biomarker for urothelial carcinoma as it enables differentiation from other genitourinary malignancies [52]. Tumor grade and invasion in biopsy material with poor morphological characteristics can be predicted as well. Thus, GATA3 represents an attractive sensitive and specific marker for detection, staging, and therapeutic management in urothelial carcinoma. In invasive urothelial carcinomas, a notable difference in GATA3 expression between the subgroups with non-muscular invasion and those with muscular invasion has been recorded [53]. Among the examined histological subtypes, the microcytic subtype demonstrated the greatest GATA3 expression correlated with better prognosis and overall survival rates [53].

### 3.4. Autoimmune Diseases

#### 3.4.1. Type 1 Diabetes (T1D)

As indicated earlier, GATA3 acts as an essential TF in controlling T-cell lineage. Since autoimmunity alters the capabilities of T cells, and T1D is one of these diseases, then GATA3 is proposed to be involved in the underlying pathogenesis of T1D as it is examined in a recent study. Deficient levels of GATA3 and forkhead box P3 (FoxP3) along with elevated T-bet and RORγt mRNA levels in individuals with positive β-cell autoantibodies were associated with T1D pathology [54]. So, considering the restoration of GATA3 levels in such T1D cases may exhibit beneficial effects for the treatment and management strategies of T1D.

#### 3.4.2. Juvenile Idiopathic Arthritis (JIA)

A novel insight into the altered GATA3 activity that coincides with autoimmune arthritis was unveiled in the first known case report of autoimmune arthritis [55]. A dominant negative function of GATA3 transcriptional activity was determined due to the expression of mutant protein M401VfsX106 [55]. It is well established that autoimmune disorders are characterized by elevated T helper 1 (Th1) and T helper 17 (Th17) cell activity, accompanied by increased Interferon-gamma (IFN-γ) release due to the epigenetic modifications; gain or loss of the histone modification and DNA methylation lead to the production of Th0, Th1, and Th2 lineages [56]. The enforcement of GATA3 expression concomitant with the reduction in Th17 cell differentiation leads to a considerable reduction in joint-destructive inflammation in arthritic mice models, indicating the protective role of GATA3 in alleviating the progression of autoimmune arthritis [57]. This discovery provides a new understanding of GATA3’s role in autoimmune diseases and potential therapeutic targets.

#### 3.4.3. Multiple Sclerosis (MS)

MS is an autoimmune disorder described by altered Th-cell differentiation that is mainly driven by GATA3 [58,59]. GATA3 is thought to decrease the onset and intensity of MS by directing the differentiation of Th cells toward Th2 and regulatory T cells (Tregs) while suppressing the differentiation of Th1 and Th17 cells. Dysfunctional Tregs, controlled by the *FoxP3* gene, contributed to the pathogenesis of MS [58,59]. Since the expression of *FoxP3* in Tregs is regulated by *GATA3*, in a retrospective case-control study, the connection among polymorphisms of both genes was evaluated and correlated to the development of MS [58]. Upon comparison, there was no significant association between FoxP3 and GATA3 polymorphisms with MS susceptibility. Nevertheless, a bioinformatic analysis identified genetic variants of GATA3 as risk factors for augmenting the incidence of relapsing-remitting multiple sclerosis (RRMS) [59]. The latter is defined as episodes or attacks of MS with different signs, symptoms, and severities. Further work is required to elaborate on the implication of GATA3 in MS pathogenesis and the potential to design GATA3-mediated treatment strategies to manage MS.

### 3.5. Neurodegenerative Diseases

#### 3.5.1. Alzheimer’s Disease (AD)

Late-onset Alzheimer’s disease (LOAD) is characterized by systemic inflammation that occurs due to dysregulated T-cell activity [60]. The cells are known for their rapid infiltration into the brain, hence aggravating the neurogenerative disease pathology. Since GATA3 represents an inevitable regulator for T-cell lineage determination, a connection between GATA3 activity and the pathogenesis of LOAD has been speculated. Network analysis detected a considerable decline in GATA3 mRNA levels in the LOAD patients’ group. Thus, molecular dysregulation in Th-related genes provided the potential for the early diagnosis or targeted interventions of AD [60]. Furthermore, genome-wide association studies (GWAS) and functional analysis detected LOAD-associated loci containing different genetic variants that were mostly present in non-coding regions in the human genome [61]. GATA3 with other proteins were detected with their corresponding risk alleles for activating HLA-DRB1 in the brains of all patients suffering from AD [61]. Prior transcriptomic analysis has shown that GATA2 and GATA3 were co-expressed with HLA-DQA1 in the AD hippocampus [62]. Taken together, GATA3 regulates AD pathology via transcriptional activation of HLA expression in the human brain that was found to be upregulated [61]. Further AD studies are recommended to focus on interfering with GATA3 expression with related proteins that might relieve symptoms, decelerate neurodegeneration, and improve AD progression and clinical outcomes.

#### 3.5.2. Parkinson’s Disease (PD)

GATA3 is a specific modulator for Th cells’ expression and activity in sympathetic nervous system (SNS) neurons [63]. T cells’ levels are diminished due to PD pathophysiology; thus, GATA3 was proposed to be involved in PD underlying mechanisms [10]. GATA3 blood levels were depleted in PD patients who experienced sleep disturbances, suggesting that GATA3 could be potentially utilized as a novel non-invasive biomarker for early detection of PD, as well as predicting individuals who are at risk of developing non-motor symptoms. In addition, the ectopic expression of GATA3 remarkably facilitated the neuronal cells’ proliferation and survival rate in PD models [10]. This finding accentuates the importance of GATA3 in neurodegenerative disease research and its potential clinical applications. Hence, tailoring GATA3-mediated treatment and management plans for PD specifically and neurodegenerative disease generally becomes plausible as it seems beneficial for patients to manage their symptoms and improve their outcomes.

### 3.6. Other Diseases

#### 3.6.1. Coronavirus Disease 2019 (COVID-19)

The prevalence of COVID-19 is increasing worldwide, and GATA3-mediated Th1 cells are the main fighters during the disease progression. Hence, GATA3 might be involved in COVID-19 dysregulated mechanisms due to the abnormal T-cell responses [64]. The previous report reinforced the severity and fatal outcomes of COVID-19 to remarkable elevations in GATA3, RORγt, and T-bet, while sharp reductions in FoxP3 expression levels were recorded [64]. Further work is required to provide a deeper insight into the role of GATA3 in the pathophysiology of COVID-19 and how it may apply to improve therapeutic approaches.

#### 3.6.2. Type 2 Diabetes (T2D)

**Figure 6:**
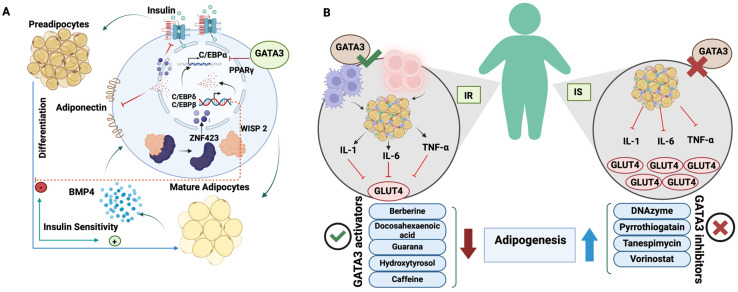
GATA3 and mediators of adipogenesis and inflammatory response in IS and IR individuals. (**A**) In mature adipocytes, BMP4 secretion initiates adipogenesis by causing the separation of WISP2 and ZNF423, enabling the translocation of ZNF423 into the nucleus. This leads to the activation of PPAR and associated factors such as C/EBPβ, δ, and α. (**B**) In IR individuals, adipose tissues exhibit elevated expression of GATA-3, which promotes the production of inflammatory cytokines, exacerbating insulin resistance. On the contrary, GATA3 levels are diminished in IS individuals, suppressing pro-inflammatory cytokines and preserving insulin sensitivity. The diagram also depicts the effects of various GATA3 activators and inhibitors on adipogenesis and inflammatory processes. IS, insulin sensitive; IR, insulin resistant; BMP4, bone morphogenetic protein 4; WISP2, Wnt1 inducible-signaling pathway protein 2; ZNF423, zinc finger protein 423; PPAR, peroxisome proliferator-activated receptors; C/EBP δ, CCAAT/enhancer-binding proteins δ; C/EBPβ, CCAAT/enhancer-binding proteins β; IL-1, interleukin-1; IL-6, interleukin 6; TNF-α, tumor necrosis factor-α; GLUT4, glucose transporter type 4. (Adapted from Al-Jaber et al. [65] and re-drawn by biorender).

Multiple earlier reports have pinpointed the role of GATA3 in obesity-related conditions that involve IR, whether left untreated or leading to the incidence of T2D. Dysfunctional adipogenesis is a significant factor in the onset of IR linked to obesity and T2D [65]. When GATA3 is silenced, adipocyte differentiation is improved, the secretion of inflammatory cytokines is properly modulated, and insulin sensitivity is restored in IR cells [65]. Consequently, GATA3 depletion represents a promising tool for alleviating the underlying pathological mechanisms associated with T2D. The beneficiary effect of inhibiting GATA3 to orchestrate the adipogenic differentiation, coordinate the release of inflammatory cytokines, and restore insulin sensitivity is summarized in Figure 6.

#### 3.6.3. Cardiovascular Diseases

**Figure 7:**
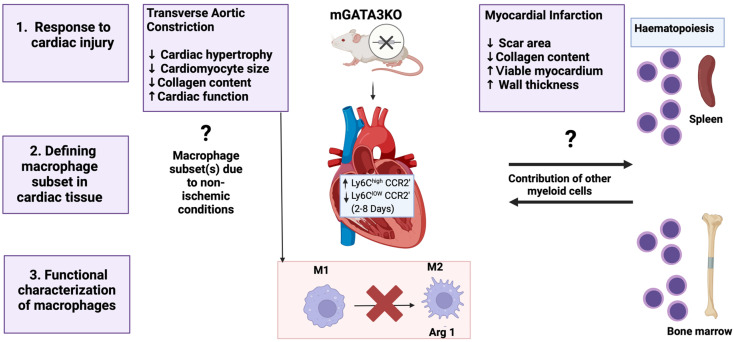
Effects of interference with mGATA3 on cardiac injury and repair. Following myocardial infarction, mGATA3 deficiency results in increased circulating Ly6ChighCCR2þ monocytes and macrophages (M1 type), along with reduced Ly6ClowCCR2 (M2 type) macrophages in the ischemic heart. mGATA3KO mice displayed cardioprotective features, likely due to the inhibition of M2 polarization of cardiac macrophages. Cardioprotective effects were also observed in a transverse aortic constriction model of cardiac pressure overload. mGATA3, GATA3 in myeloid cells; mGATA3KO, knockout of GATA3 in myeloid cells; BM, bone marrow. (Adapted from Giannarelli et al. [67] and re-drawn by biorender).

Despite the lack of research focusing on the role of GATA3 in the pathogenesis and complications of cardiovascular diseases, a prior study has shed light on the significance of dampening GATA3 in improving cardiac function following myocardial infarction (MI) and pressure overload hypertrophy [66]. As GATA3 is not normally found in healthy cardiac tissues, it accumulates in the myocardium after MI injury, exacerbating the pathology [66]. Given that monocytes are the primary source of myocardial macrophage in acute MI, a build-up of GATA3 most likely occurred in monocyte-derived macrophages. Dampening GATA3 levels within these subsets of macrophages was associated with enhanced cardiac function in knockdown mice models (Figure 7) [66,67]. The latter finding provides insight into designing useful immunotherapeutic approaches involving GATA3 signaling in macrophages to treat cardiac diseases.

## 4. GATA3 Is a Potential Therapeutic Target

### 4.1. Molecular Mechanisms and Therapeutic Implications

#### 4.1.1. GATA3 Pathways for Cancer Therapy

The designation of therapeutic targets for cancer treatment and disease management is challenging due to the heterogeneity in tumor types, origin, epigenetic modifications, TME, and metastatic sites [68]. Accordingly, GATA3 is differentially expressed in different tumor types and stages; therefore, targeting GATA3 for cancer therapy is variable [1]. Moreover, identifying resistance mechanisms that may develop in certain cancers is crucial as these mechanisms may contribute to the limited or lack of response to certain treatment modalities, resulting in recurrent tumor growth [69].

GATA3 expression has been linked to improved prognosis in BC. However, a study featuring the alteration in gene signatures in BC patients led to a mutational shift in GATA3 causing the switch from an anti-tumoral to a pro-tumoral phenotype altering the overall survival rates in BC patients [32]. Hence, mechanisms relying on GATA3 may require specific attention as the patient stratification based on GATA3 behavior may add a significant layer in therapeutic considerations for accurate prognosis and treatment plans [32]. Evidence indicated the association between GATA3 expression and the reversal of EMT that dampens tumor metastasis. The molecular mechanisms underlying the selection of GATA3 upregulation in well-differentiated epithelium-like BCs rather than invasive cancers have been elucidated so far [70]. Therefore, GATA3 displays a non-invasive diagnostic and prognostic biomarker for BC. Noticeably, the necessity of determination of GATA3 expression in BC is not limited to tumor stratification and molecular subtyping, as it is crucial for therapeutic designation and combination treatment plans. Prior research has revealed that BC tumors with high GATA3 levels will not respond to doxorubicin treatment as GATA3 contributes to resistance mechanisms [71]. GATA3 promotes cell viability and survival by reducing the expression of the ferroptosis-associated gene *CYB5R2*, which in turn maintains iron homeostasis. Therefore, *GATA3* and *CYB5R2* are directly associated with response to neoadjuvant chemotherapy (NAC) considered the primary preoperative therapy for BC [71].

Emerging evidence has discussed the implication of genetic variants of GATA3 in the development and progression of leukemia. A genetic polymorphism of GATA3 called the rs3824662 risk allele was linked to a higher risk of relapse and inferior outcome in leukemic patients [72,73]. Through bioinformatics analyses and cellular experiment validations, potential GATA3-related genes, multiple signaling pathways (involved in T- and B-cell leukemogenesis), and the risk allele of *GATA3* SNP have been unveiled that in turn increase the feasibility of targeting GATA3 for prognostic and management strategies in leukemia [74]. The feasibility of targeting GATA3 for treatment strategies is completely reliable in comprehending the fundamental processes and clinical condition of each disease that alter the GATA3 expression levels and activity. Further investigations are required so far to provide a deeper insight into targeting GATA3 as novel therapeutic designations that might move from bench to bedside to benefit patients.

#### 4.1.2. Modulating GATA3 for Therapeutic Interventions in Autoimmune Diseases

GATA3 serves as a key regulator of immune balance and T-cell activity. Since the regulatory T cells (Tregs) that are governed by the Foxp3 gene play a protective role against autoimmunity, a connection between GATA3 and the regulation of Treg activity is likely mediated through GATA3’s modulation of Foxp3 expression [2]. Notably, GATA3 exhibits a dual function in both the progression and control of autoimmune diseases. Reduced GATA3 levels, for instance, have been linked to the exacerbation of T1D pathology. Although lowered GATA3 levels were found to aggravate T1D pathology, a contradictive study revealed the detrimental consequences of elevated GATA3 expression on the progression of T1D [75]. The elevation of GATA3 altered the subpopulation of Tregs found in the islet cells, causing their dysfunction, compromising their function, and contributing to T1D onset [75]. Furthermore, targeting GATA3 mutations associated with JIA is a potential strategy to alleviate symptoms and improve patients’ outcomes [55]. As Th2 cells, stabilized by GATA3 activity, provide neuroprotection for MS disease, then GATA3 overexpression, stimulating a Th2 response, can reduce disease severity and delay the onset of experimental autoimmune encephalomyelitis (EAE) in an MS animal model [76]. These findings could pave the way for future translational studies in MS via exploring genetic biases toward different Th subsets influencing the clinical and histological patterns of demyelinating diseases.

#### 4.1.3. Exploring GATA3 Regulation for Neurodegenerative Therapy

Bioinformatics analysis of transcriptomic data is widely used to identify molecular signatures and assign therapeutic targets for various conditions. GATA3 has been highlighted in the regulatory signature of vascular dementia (VaD), raising the potential of targeting GATA3 for therapeutic interventions assignment as neurodegenerative conditions [77]. Considering the beneficial role of GATA3 in regulating AD pathology via transcriptional activation of HLA61, thereby GATA3 serves as a promising target for managing dementia-associated conditions [77]. Since GATA3 was depleted in PD patients who experienced sleep disturbances, then, its ectopic expression remarkably facilitated the neuronal cells’ proliferation and survival via transcriptional inhibition of the transient receptor potential melastatin 2 (TRPM2) [10,78]. Consequently, the measurement of GATA3 expression in blood could be a potential novel biomarker for diagnosing idiopathic Parkinson’s disease (PD) and assessing the severity of the condition [10].

#### 4.1.4. GATA3 Role for the Management of Type 2 Diabetes

Given that GATA3 silencing prevented fat accumulation, decreased inflammation, and restored insulin sensitivity, targeting GATA3 demonstrates a promising tool for relieving T2D-associated symptoms [65].

#### 4.1.5. GATA3 Therapeutic Implications in Cardiovascular Disease

GATA3 is not normally expressed in healthy cardiac tissues but usually accumulates in monocyte-derived macrophages in response to myocardial infarction (MI) and pressure overload hypertrophy [66]. Collectively, targeting GATA3 expresses an auspicious tool for recovery from cardiac injury.

### 4.2. Challenges and Limitations

While targeting GATA3 presents a promising therapeutic strategy for various diseases, several challenges and limitations must be addressed. The development of effective delivery systems, the specificity of gene silencing, and the potential side effects are critical factors that need careful consideration. Further research and clinical trials are essential to validate these approaches and translate them into effective treatments for patients.

### 4.3. Feasible Strategies for GATA3 Therapeutic Targeting

#### 4.3.1. Delivery Mechanisms: siRNA and DNAzyme Delivery

Recent advancements have explored the use of small interfering RNAs (siRNAs) and DNAzymes to knock down GATA3 expression. For instance, pulmonary delivery of siRNAs using polyethylenimine (PEI)-based carriers has shown promise in targeting GATA3 in activated T cells for the treatment of allergic asthma. However, stability and targeting issues remain significant hurdles in addition to the complicated process of T cells’ transfection [79,80].

#### 4.3.2. Post-Translational Modifications: Acetylation and DNA Binding

GATA3’s function is regulated by post-translational modifications, such as acetylation by the histone acetyltransferase p300. Acetylation at specific residues (e.g., K358) is required for optimal DNA binding. Targeting the GATA3/p300 complex or modulating GATA3’s acetylation state presents a novel therapeutic approach, but further research is needed to develop effective inhibitors [44,81].

### 4.4. Potential Side Effects

#### 4.4.1. Off-Target Effects: Specificity of Gene Silencing

The use of siRNAs and DNAzymes to silence GATA3 expression must be carefully designed to avoid off-target effects [79,80,82]. Non-specific gene silencing can lead to unintended consequences, affecting other critical pathways and causing adverse effects [83].

#### 4.4.2. Immune Response: Immune Activation

Modulating GATA3 expression can impact the immune system, given its role in T-cell differentiation and function. For example, targeting GATA3 in T cells could potentially alter immune responses, leading to increased susceptibility to infections or autoimmune reactions [2,79,84,85].

### 4.5. Current Status of GATA3-Targeted Therapies

#### 4.5.1. Preclinical Studies: siRNA and DNAzyme Delivery Systems

Preclinical studies have demonstrated the potential of siRNA and DNAzyme delivery systems in targeting GATA3. For instance, Tf-Mel-PEI polyplexes have shown effective GATA3 silencing in lung slices, indicating their feasibility for treating diseases like asthma. However, these approaches are still in the experimental stage and require further validation in clinical settings [79,80].

#### 4.5.2. Clinical Trials

Currently, there are limited clinical data on GATA3-targeted therapies. Most research is still in the preclinical phase, and more studies are needed to evaluate the safety, efficacy, and long-term effects of these therapies in humans.

#### 4.5.3. Combination Therapies

Combining GATA3-targeted therapies with other treatments, such as immune checkpoint inhibitors or chemotherapy, may enhance therapeutic outcomes. For example, targeting GATA3 in combination with other pathways involved in cancer progression could provide a more comprehensive approach to treatment [32,86].

## 5. Future Directions and Conclusions

### 5.1. Emerging Areas of GATA3 Research

Developing research on GATA3 is offering insights for novel GATA3 contributions beyond traditional immune system functions to unravel its implications in health and disease-related processes. Recent studies are exploring GATA3’s expressions in cancer biology, where it is potentially used as a biomarker for diagnosis and prognosis in cancers like breast and prostate cancer. In neurobiology, researchers are investigating GATA3’s importance for neuronal development and survival, with potential applications in neurodegenerative diseases such as Parkinson’s disease [10]. GATA3’s impact on metabolic disorders is being evaluated as well, particularly for adipogenesis and insulin sensitivity, which potentiates novel therapies for obesity and type 2 diabetes [65]. Additionally, there is an increasing interest in the elucidation of the GATA3 regulatory mechanisms of stem cell biology and developmental processes, which includes its functions in tissue regeneration and embryonic development [87,88]. These new research areas suggest that GATA3 is a versatile transcription factor with broad implications for both basic research and clinical applications.

### 5.2. Implications of Novel Findings

#### 5.2.1. Enhanced Diagnostic and Prognostic Tools

The identification of GATA3 as a sensitive diagnostic marker for various cancers, including breast cancer and urothelial carcinoma, enhances the accuracy of disease diagnosis and prognosis [28,53]. This can lead to better patient stratification and personalized treatment plans.

#### 5.2.2. Targeted Therapeutic Strategies

Understanding the molecular mechanisms regulating GATA3 expression and activity opens new avenues for targeted therapies. For example, targeting the ZPO2/GATA3 signaling axis in breast cancer [39] or modulating GATA3-AS in liver carcinoma [46] could provide effective treatment options.

#### 5.2.3. Biomarker Development

The potential use of GATA3 as a novel biomarker for early detection of diseases like Parkinson’s disease [10] and its role in predicting disease progression describes its importance in developing non-invasive diagnostic tools.

#### 5.2.4. Research and Clinical Applications

These findings encourage further research into the regulatory mechanisms of GATA3 and its interactions with other molecular pathways. This can lead to the development of novel therapeutic interventions and improve clinical outcomes for patients with GATA3-related diseases.

## 6. Concluding Remarks

In summary, GATA3 stands out as a principal transcription factor with diverse roles in health and disease. Its intricate implication in developmental processes and tissue-specific functions underscores its indispensability in maintaining physiological homeostasis across multiple tissues and organ systems, including the immune system, mammary glands, kidneys, and central nervous system. The dysregulation of GATA3 is implicated in a wide spectrum of diseases, ranging from cancers, including breast, prostate, and T-cell lymphomas, to autoimmune and neurodegenerative disorders. These associations state GATA3’s dual role as a biomarker for disease prognosis and a potential therapeutic target. Exploring GATA3-related molecular mechanisms has unraveled its primary involvement in cellular signaling pathways and regulatory networks. Overall, the discovery of new facets of GATA3 in cellular and tissue biology provides novel insights for promising avenues of therapeutic interventions focusing on restoring GATA3 functionality in disease states. Consequently, the identification of personalized medicine strategies for conditions influenced by GATA3 dysregulation becomes more feasible to improve overall patient outcomes.

## Data Availability

Not applicable.
